# Supplementary material for: Sero-prevalence of SARS-CoV-2 antibodies in Ethiopia: Results of the National Population Based Survey, 2021
Source: PLoS One. 2025 May 6;20(5):e0313791. doi: 10.1371/journal.pone.0313791 (PMC12054855; doi:10.1371/journal.pone.0313791)
Supplement: Attachment — Submitted filename: Response_to_Reviewers_auresp_2.docx [file pone.0313791.s003.docx]

**Nov 24 2024**

**PONE-D-24-10224** **PONE-D-24-10224R1**

**Manuscript Title: Sero-prevalence of SARS-CoV-2 antibodies in Ethiopia: Results of the National Population Based Survey, 2021**

Dear Editor,

On behalf of the authors, and myself I would like to thank you for and the reviewers for having critically reviewed the manuscript and for giving allowing us to revise and re-resubmit it to PLOS ONE. We have revised the manuscript based on the comments and suggestions from you and all three reviewers.

We deeply appreciate yours and the reviewers' comments and suggestions, which we found very helpful and used to improve the quality of the manuscript. Per your advice, we have prepared a point-by-point response to all comments as presented below. We have also made changes as advised in a separate file titled 'Revised Manuscript with Track Changes' and have uploaded the file per PLOS guidelines. In addition, we have uploaded three documents as part of our online submission: the tracked and clean copies of the manuscript as well as a rebuttal letter.

Sincerely

Birra Bejiga

Reviewer's Responses to Questions

**Comments to the Author**

1. If the authors have adequately addressed your comments raised in a previous round of review and you feel that this manuscript is now acceptable for publication, you may indicate that here to bypass the “Comments to the Author” section, enter your conflict of interest statement in the “Confidential to Editor” section, and submit your "Accept" recommendation.

Reviewer #1: All comments have been addressed

Reviewer #2: (No Response)

Reviewer #4: (No Response)

2. Is the manuscript technically sound, and do the data support the conclusions?

The manuscript must describe a technically sound piece of scientific research with data that supports the conclusions. Experiments must have been conducted rigorously, with appropriate controls, replication, and sample sizes. The conclusions must be drawn appropriately based on the data presented.

Reviewer #1: Yes

Reviewer #2: (No Response)

Reviewer #4: Partly

3. Has the statistical analysis been performed appropriately and rigorously?

Reviewer #1: Yes

Reviewer #2: (No Response)

Reviewer #4: No

4. Have the authors made all data underlying the findings in their manuscript fully available?

The [PLOS Data policy](http://www.plosone.org/static/policies.action#sharing) requires authors to make all data underlying the findings described in their manuscript fully available without restriction, with rare exception (please refer to the Data Availability Statement in the manuscript PDF file). The data should be provided as part of the manuscript or its supporting information, or deposited to a public repository. For example, in addition to summary statistics, the data points behind means, medians and variance measures should be available. If there are restrictions on publicly sharing data—e.g. participant privacy or use of data from a third party—those must be specified.

Reviewer #1: Yes

Reviewer #2: (No Response)

Reviewer #4: No

5. Is the manuscript presented in an intelligible fashion and written in standard English?

PLOS ONE does not copyedit accepted manuscripts, so the language in submitted articles must be clear, correct, and unambiguous. Any typographical or grammatical errors should be corrected at revision, so please note any specific errors here.

Reviewer #1: Yes

Reviewer #2: (No Response)

Reviewer #4: Yes

6. Review Comments to the Author

Please use the space provided to explain your answers to the questions above. You may also include additional comments for the author, including concerns about dual publication, research ethics, or publication ethics. (Please upload your review as an attachment if it exceeds 20,000 characters)

**Reviewer #1:**  All the concerns have been addressed in all sections of manuscript, including sample size, sampling and recommendation.

**Authors’ response:** Thanks. Noted.

**Reviewer #2****:** (No Response):

**Reviewer #4**: Abstract
The statistical methodology of this study involved descriptive analysis, sampling using a complex survey design, and mixed-effects logistic regression analysis.

**Authors’ response:** Thanks. Noted.

The descriptive analysis requires a table presenting the demographic and clinical characteristics of the study sample. It is important to note the distinction between the study sample and the broader sampled population. The current Table 1 describes the overall population, but no table provides a descriptive analysis of the study sample. This information is important to understand the sizes of subgroups analyzed in the logistic regression.

**Authors’ response:**

**Many thanks for your reflection. Lines 611 Table 1, Column 3, labelled ‘Pop. Distribution' indicates the weighted percentage distribution of the demographic and clinical variables. This approach is the recommended data analysis presentation, as it adjusts for survey weights, rather than simply displaying descriptive statistics without such adjustment.** **However, we revised it with some correction.**

Another concern with Table 1 is that it combines seroprevalence levels estimated in the study with data on the national population obtained from the census. I strongly recommend separating these into two distinct tables. This will give a clear notion on which results are from the current study. Additionally, since the table on seroprevalence contains the same information as Figure 1, I say that Figure 1 should be removed. While Figure 1 is visually appealing, a table would be more useful for readers who need exact numerical estimates.

**Authors’ response:**

**Thank you for your critical review. As per the reviewer's suggestion, lines 593 revised as suggested we have removed Figure 1, as it depicts similar information to that presented in Table 1. However we did not use census data in this analysis; instead, we applied survey weights to the sample of 12,756, which is crucial for adjusting of over and under sampling estimation. This sample represents a population of 19,456,360 in Ethiopia through the application of survey weights. Therefore, we combined both pieces of information into a single table, as suggested.**

The estimation of the sample size is included in this version of the manuscript, but there are issues with the formula provided. The formula does not include a term representing the required level of confidence (usually expressed as a function of the z statistic). Furthermore, the factor accounting for prevalence is conventionally expressed as p(1-p) , but the formula in the manuscript uses (1/p - 1) . Therefore, this should be verified and have an explanation in a response. It is also worth noting that the current formatting makes it appear as though the "-1" is an exponent of p , which could be misleading.

**Authors’ response:**

**Line 163, revised and added as suggested.** However, the sample size calculation formula we used was the national sample size determination formula **proposed and utilized by the Demographic and Health Survey group**. This formula is derived from the p (1−p) formula, which is suitable for national surveys and particularly favours for small p values. The p (1−p) formula is mostly applicable for small-scale surveys and single population proportions.

Additionally, applying the formula with the numbers provided yields a sample size estimate of N = 21,495, which differs from the value reported in the manuscript. Again, more clarification is required.

**Authors’ response:** Point well taken. We made the necessary corrections and adjustments to the sample size and sample size formula the details are now given on (lines 141-172) the confusion arose unintentionally. We apologize for any confusion this has created.

Finally, the logistic regression appears to be well conducted, and the results are presented effectively. However, it remains critical to include a table showing the frequencies (descriptive analysis) of the variables analyzed in the obtained sample. This would allow for the identification of potential biases in certain groups, which should be acknowledged in the Discussion section, if biases arise.

**Authors’ response:**

Thank you Point well taken. However, it was indicated on table 1 column 3 on (line 612) the weighted percentage distribution of the demographic and clinical variables. We combine both the descriptive statistics for each variable and the corresponding SARS-CoV-2 prevalence into one Table to reduce the number of Tables.

Additional comments below.

Abstract

Several sections of the text use "COVID-19" and "SARS-CoV-2" almost interchangeably. It is important to make distinction between the disease and the virus. Therefore, phrases such as “seroprevalence of COVID-19,” “infected by COVID-19,” and “spread of COVID-19” need revision to ensure the correct terminology is used throughout the manuscript.

**Authors’ response: thank you revised as suggested.**

Additionally, the name of the disease, COVID-19, should always be capitalized and used consistently across the text.

**Authors’ response:** **thank you corrected and edited.**

In the Abstract, it is stated that AOR (adjusted odds ratios) were used to identify independent associations. However, it might be more accurate to describe the findings as identifying groups at higher risk of having been infected at least once.

**Authors’ response: Thank you, revised and added as suggested.**

The reported general seroprevalence levels differ slightly between the Abstract and the Results section. I recommend verifying these values and ensuring consistency throughout the manuscript to avoid any discrepancies.

**Authors’ response: This is now corrected and well in order**

Introduction

- Sentences appear truncated in lines 85–88.

**Authors’ response:** This is now complete

- Line 90 (sensitivity of the test): Specify which test is being referred to in this context.

**Authors’ response: thank you Revised as suggested.**

Methods

- Define "HH" upon first use.

**Authors’ response: thank you Revised as suggested.**
- Line 167 : Replace d with \delta.

**Authors’ response: Thank you replaced and edited as suggested.**

- Clarify how the design effect (DEFT) was estimated at 2.25.

**Authors’ response:** Many thanks we have identified that it was not correctly wrote. This in now corrected and it all makes sense.

- As mentioned, provide more explanation on the sample size formula and verify it.

**Authors’ response:**

The sample size formula was taken from the Demographic and Health survey manual which is developed from the usual sample size formula p(1-p) that is planned for national sample size determination to favor the small proportions.

Results

- As mentioned earlier, a table presenting the demographic and clinical information of the study sample should be included.

**Authors’ response:** Thank you Point well taken. However, it was indicated on table 1 column 3 on (line 612) the weighted percentage distribution of the demographic and clinical variables. We combine both the descriptive statistics for each variable and the corresponding SARS-CoV-2 prevalence into one Table to reduce the number of Tables.

- As previously noted, Figure 1 should be removed since the seroprevalence data it displays is already provided in a table.

**Authors’ response: Deleted** as suggested

- If the proportion of individuals with no exposure to either IgG or IgM is reported as 90.7%, then the exposure rate should be 9.3%. However, the abstract states this as 10%. This discrepancy needs to be addressed to ensure clarity and consistency throughout the manuscript, including in the abstract.

**Authors’ response:** This is reviewed we have identified that it was not consistence. This in now corrected and it all makes sense.

- Table 3: phrase "ever sicked" requires correction.

**Authors’ response:** Done

Discussion

- I think that finding non-significant results about household size requires discussion. This is yet another reason why the table with characteristics of the sample is important.

**Authors’ response: Noted**

7. PLOS authors have the option to publish the peer review history of their article ([what does this mean?](https://journals.plos.org/plosone/s/editorial-and-peer-review-process#loc-peer-review-history)). If published, this will include your full peer review and any attached files.


If you choose “no”, your identity will remain anonymous but your review may still be made public.


**Do you want your identity to be public for this peer review?** For information about this choice, including consent withdrawal, please see our [Privacy Policy](https://www.plos.org/privacy-policy).

Reviewer #1: **Yes:** Sumanth Mallikarjuna Majgi

Reviewer #2: **Yes:** Prince Obinna Anyanwu

Reviewer #4: No

[NOTE: If reviewer comments were submitted as an attachment file, they will be attached to this email and accessible via the submission site. Please log into your account, locate the manuscript record, and check for the action link "View Attachments". If this link does not appear, there are no attachment files.]

While revising your submission, please upload your figure files to the Preflight Analysis and Conversion Engine (PACE) digital diagnostic tool, <https://pacev2.apexcovantage.com/>. PACE helps ensure that figures meet PLOS requirements. To use PACE, you must first register as a user. Registration is free. Then, login and navigate to the UPLOAD tab, where you will find detailed instructions on how to use the tool. If you encounter any issues or have any questions when using PACE, please email PLOS at <a href="mailto:figures@plos.org">figures@plos.org. Please note that Supporting Information files do not need this step.

*In compliance with data protection regulations, you may request that we remove your personal registration details at any time.* [*(Remove my information/details)*](https://www.editorialmanager.com/pone/login.asp?a=r)*. Please contact the publication office if you have any questions.*
