# Supplementary material for: Sero-prevalence of SARS-CoV-2 antibodies in Ethiopia: Results of the National Population Based Survey, 2021
Source: PLoS One. 2025 May 6;20(5):e0313791. doi: 10.1371/journal.pone.0313791 (PMC12054855; doi:10.1371/journal.pone.0313791)
Supplement: Attachment — Submitted filename: Response to Reviewers.docx [file pone.0313791.s002.docx]

**August 13, 2024**

**PONE-D-24-10224**

**Manuscript Title: Sero-prevalence of SARS-CoV-2 antibodies in Ethiopia: Results of the National Population Based Survey, 2021**

Dear Editor,

On behalf of the authors, and myself I would like to thank you for and the reviewers for having critically reviewed the manuscript and for giving allowing us to revise and re-resubmit it to PLOS ONE. We have revised the manuscript based on the comments and suggestions from you and all three reviewers.

We deeply appreciate yours and the reviewers' comments and suggestions, which we found very helpful and used to improve the quality of the manuscript. Per your advice, we have prepared a point-by-point response to all comments as presented below. We have also made changes as advised in a separate file titled 'Revised Manuscript with Track Changes' and have uploaded the file per PLOS guidelines. In addition, we have uploaded three documents as part of our online submission: the tracked and clean copies of the manuscript as well as a rebuttal letter.

Sincerely

Birra Bejiga

Journal Requirements:

When submitting your revision, we need you to address these additional requirements.

1. Please ensure that your manuscript meets PLOS ONE's style requirements, including those for file naming. The PLOS ONE style templates can be found at.

**Authors’ response:** We have double-checked and ensured that all PLOS ONE’s style requirements are met

2. Please provide additional details regarding participant consent. Since your study included minors, state whether you obtained consent from parents or guardians. If the need for consent was waived by the ethics committee, please include this information

**Authors’ response:** Written informed consent was obtained from each survey participant for the interview and blood sample collection, and for testing and the detailed description is in the method section

3. We note that you have indicated that there are restrictions to data sharing for this study. For studies involving human research participant data or other sensitive data, we encourage authors to share de-identified or anonymized data. However, when data cannot be publicly shared for ethical reasons, we allow authors to make their data sets available upon request. For information on unacceptable data access restrictions, please see http://journals.plos.org/plosone/s/data-availability#loc-unacceptable-data-access-restrictions.

Before we proceed with your manuscript, please address the following prompts:

a) If there are ethical or legal restrictions on sharing a de-identified data set, please explain them in detail (e.g., data contain potentially sensitive information, data are owned by a third-party organization, etc.) and who has imposed them (e.g., an ethics committee). Please also provide contact information for a data access committee, ethics committee, or other institutional body to which data requests may be sent.

**Authors’ response:** There are no ethical and legal restrictions regarding sharing our data. In our data, there is no such information that is sensitive to any particular community. The contact information of the ethical review committee is the Scientific and Ethical Review Office (SERO):

***https://ephi.gov.et/about-us/departments-units/scientific-and-ethical-review-office-sero/***

National data manager center*:* ***https://ndmc.ephi.gov.et/***

Directorate of HIV and TB of Ethiopian Public Health Institute:

***https://ephi.gov.et/research/hiv-and-tb/***

  b) If there are no restrictions, please upload the minimal anonymized data set necessary to replicate your study findings as either Supporting Information files or to a stable, public repository and provide us with the relevant URLs, DOIs, or accession numbers. For a list of acceptable repositories, please see http://journals.plos.org/plosone/s/data-availability#loc-recommended-repositories.

We will update your Data Availability statement on your behalf to reflect the information you provide.

**Authors’ response:** Due to confidentiality agreements, data can be made available subject to a non-disclosure agreement. For further information, you may contact the national data manager canter of the Ethiopian Public Health Institute at ***https://ndmc.ephi.gov.et.***

4. We note that Figures 2, 3 , and 4 in your submission contain [map/satellite] images which may be copyrighted. All PLOS content is published under the Creative Commons Attribution License (CC BY 4.0), which means that the manuscript, images, and Supporting Information files will be freely available online, and any third party is permitted to access, download, copy, distribute, and use these materials in any way, even commercially, with proper attribution. For these reasons, we cannot publish previously copyrighted maps or satellite images created using proprietary data, such as Google software (Google Maps, Street View, and Earth). For more information, see our copyright guidelines: http://journals.plos.org/plosone/s/licenses-and-copyright.

We require you to either (1) present written permission from the copyright holder to publish these figures specifically under the CC BY 4.0 license, or (2) remove the figures from your submission:

a. You may seek permission from the original copyright holder of Figures 2, 3 , and 4 to publish the content specifically under the CC BY 4.0 license.
b. If you are unable to obtain permission from the original copyright holder to publish these figures under the CC BY 4.0 license or if the copyright holder’s requirements are incompatible with the CC BY 4.0 license, please either i) remove the figure or ii) supply a replacement figure that complies with the CC BY 4.0 license. Please check copyright information on all replacement figures and update the figure caption with source information. If applicable, please specify in the figure caption text when a figure is similar but not identical to the original image and is therefore for illustrative purposes only.

**Authors’ response:** The maps included in the manuscript were created using geocoded data collected with a tablet at the location of the households in a field. All geocoded data was aggregated to the regional level and presented on the corresponding map. The shape files for the maps were obtained from the online source at ***https://data.humdata.org/dataset/cod-ab-eth***

Reviewer's Responses to Questions

**Comments to the Author**

1. Is the manuscript technically sound, and do the data support the conclusions?

The manuscript must describe a technically sound piece of scientific research with data that supports the conclusions. Experiments must have been conducted rigorously, with appropriate controls, replication, and sample sizes. The conclusions must be drawn appropriately based on the data presented.

Reviewer #1: Yes

Reviewer #2: Yes

Reviewer #3: Partly

2. Has the statistical analysis been performed appropriately and rigorously?

Reviewer #1: No

Reviewer #2: Yes

Reviewer #3: No

3. Have the authors made all data underlying the findings in their manuscript fully available?

The PLOS Data policy requires authors to make all data underlying the findings described in their manuscript fully available without restriction, with rare exception (please refer to the Data Availability Statement in the manuscript PDF file). The data should be provided as part of the manuscript or its supporting information, or deposited to a public repository. For example, in addition to summary statistics, the data points behind means, medians and variance measures should be available. If there are restrictions on publicly sharing data—e.g. participant privacy or use of data from a third party—those must be specified.

Reviewer #1: No

Reviewer #2: Yes

Reviewer #3: Yes

4. Is the manuscript presented in an intelligible fashion and written in standard English?

PLOS ONE does not copyedit accepted manuscripts, so the language in submitted articles must be clear, correct, and unambiguous. Any typographical or grammatical errors should be corrected at revision, so please note any specific errors here.

Reviewer #1: Yes

Reviewer #2: Yes

Reviewer #3: Yes

5. Review Comments to the Author

Please use the space provided to explain your answers to the questions above. You may also include additional comments for the author, including concerns about dual publication, research ethics, or publication ethics. (Please upload your review as an attachment if it exceeds 20,000 characters)

**Reviewer #1:**  The study is important for decision making of the vaccine strategy and also understanding of the disease. i congratulate teh authors.

**Authors’ response:** Thanks. Noted.

However, it is expected to provide following clarifications

I. Sample size estimation is not clearly mentioned

**Authors’ response:** These are now addressed in detail under subtopics, Sample size and sampling procedure.

II. What was the random selection method for individual selection within household?

**Authors’ response:** Random selection is a form of sampling where a representative group of research participants is selected from a larger group by chance. this is now described under (line 297-328).

III. How much non-response was considered?

**Authors’ response:** Paragraph (lines 176-179) revised non-response rate is included.

IV. Statistical methods adopted is not complete

**Authors’ response:** Paragraph (lines 223-247) revised as suggested under the Statistical analysis part.

V. No multivariate analysis is done, it is better to do that and discuss the findings. If not done for any particular reason, better to discuss and justify them.

**Authors’ response:** These are now addressed in detail under topics: Factors associated with COVID-19 Seroprevalence Results of a multilevel mixed effect logistic regression model

(lines 191-228).

**Reviewer #2****:** This is an important study because if it is the first population-based inquiry in Ethiopia. The manuscript is written in a way that makes for ease of understanding. However, I have the following observations/suggestions:

**Authors’ response:** Thanks. Noted.

A. line66-69: Link the sentences together to the citation

**Authors’ response:** Done

B. line 70: Punctuation at the end of the sentence

**Authors’ response: Done**

C. line89-94: link sentences to citation

**Authors’ response: Done**

D. 268-270: I suggest you add prevalence figure to the age. This is will aid understanding.

**Authors’ response: this is now** added and corrected

E. Line 290: The statement "nearly one in ten" is difficult to comprehend. You may like to use actual prevalence here.

**Authors’ response:** This is now corrected and well in order.

F. I understand the style of putting the tables and figures as appendages, but I suggest putting them in the body og the writing, unless the will be highlighted as links.

**Authors’ response:** Formatted as suggested and cross-referenced by highlighting the link

**Reviewer #3**: Abstract
The writers have properly captured the essence of the work by highlighting the key research question, the method(s) used and their key findings in a style that is clear, easy to read and comprehend.

**Authors’ response:** Thanks. Noted.

 Concerns:
1. Conclusion and recommendation: You already have your result from the study, rather than draw your conclusion on "the limited knowledge on SARSCoV-2 seroprevalence", connect your conclusion to the prevalence levels observed in your study and the implications of same.

**Authors’ response: The conclusion is revised. The Point is well taken.**

2. What is the antibodies majority of your population as you indicated on line 52 of your submission?
 **Authors’ response:** Point well taken. We revised the Conclusion and recommendation section addressing the comments given here and by others.

Introduction
The writers provide a very good background on the subject including highlighting some interesting hypothesis on why the pandemics spared Africa as well as the a good argument for using serosurveys to determine the true extent of the pandemic considering the use-case limitation to the RT-PCR...how it misses asymptomatic and mildly symptomatic people who may choose not to attend the hospital for care or may not be considered for testing with RT-PCR at the triage.

**Authors’ response:** Thanks. Noted.

**Concerns**:

1. In lines 68 and 69, you need to specifically say that the number of REPORTED SARSCoV-2 infections could be underestimating the true number of infections sin your study population

**Authors’ response:** This is reviewed. We have fixed it now that we realized it needed to be connected to previous sentences and cited. Now corrected.

2. Also, I feel that the conclusive part of your introduction could help recast the conclusion in your abstract to relate more to your findings and its implications

**Authors’ response:** The conclusion section revised taking this comment and other to shorten and make the section crisper.

Methodology
concerns
1. what is the justification for using 16years as the lower boundary for age in your study?

**Authors’ response**: Published serological studies suggest that younger adults (particularly those aged above 15y) often have high cumulative rates of SARS-CoV-2 infection in the community. The covid-19 infection is much less in age less than 15 due to different scenarios. We undertook a literature review using the Living Evidence on COVID-19, a database collecting COVID-19 related published articles from Pubmed and EMBASE and preprints from medRxiv and bioRxiv, with MESH terms.

2. On line 133, confirm which EA size parameter was used in the first stage. Was it HH-size, HH-number, land mass? You need to be specific.

**Authors’ response:** Paragraph (lines 133) revised as suggested.

3. On line 154-6: Since the category of staff that conducted the RDT screening is not explicitly stated, is it safe to assume that the Data collectors and field supervisors who were trained are the ones who did the testing? Please what is their professional qualification?

**Authors’ response:** These are now addressed in detail under subtopics data collection and participant consent.

4. Were the test performance characteristics of the RDT kit verified before deployed for this study?

**Authors’ response:** Quality control (QC) panels consisting of a positive and negative control specimen were done in parallel with the testing procedure to ensure test kits were performing correctly**.** This is now indicated in the manuscript (lines 210-219).

5. How did you obtain written consent from all participants, were they all able to read and sign the document?
 **Authors’ response:** Unfortunately, the majority of our study participants are literate; nevertheless, for those who are not, we followed the instructions for Illiterate Subjects while presenting the informed consent form orally.

Result and Discussion
1. State the actual P values where statistical tests were done and for all instance where differences were observed such as region, age etc please state the actual p value rather than "P value 0.05".

**Authors’ response:** Point well taken. Revised the result and discussion section addressing the comments given here and by others.

2. Discuss implication of the SARSCoV2 IgM observation in your study

**Authors’ response: Done**

6. PLOS authors have the option to publish the peer review history of their article (what does this mean?). If published, this will include your full peer review and any attached files.


If you choose “no”, your identity will remain anonymous but your review may still be made public.


**Do you want your identity to be public for this peer review?** For information about this choice, including consent withdrawal, please see our Privacy Policy.

Reviewer #1: **Yes:** Sumanth Mallikarjuna Majgi

Reviewer #2: **Yes:** Prince Obinna Anyanwu

Reviewer #3: No

[NOTE: If reviewer comments were submitted as an attachment file, they will be attached to this email and accessible via the submission site. Please log into your account, locate the manuscript record, and check for the action link "View Attachments". If this link does not appear, there are no attachment files.]

While revising your submission, please upload your figure files to the Preflight Analysis and Conversion Engine (PACE) digital diagnostic tool, https://pacev2.apexcovantage.com/. PACE helps ensure that figures meet PLOS requirements. To use PACE, you must first register as a user. Registration is free. Then, login and navigate to the UPLOAD tab, where you will find detailed instructions on how to use the tool. If you encounter any issues or have any questions when using PACE, please email PLOS at <a href="mailto:figures@plos.org">figures@plos.org. Please note that Supporting Information files do not need this step.

*In compliance with data protection regulations, you may request that we remove your personal registration details at any time. (Remove my information/details). Please contact the publication office if you have any questions.*

*With Best Regards*

*Birra Bejiga*

*MPH With Specialty in Epidemiology and Biostatistics.*

*Researcher at Ethiopian public health institute (EPHI) ,*

*HIV TB Research Directorate, HIV surveillance team.*

*Email: birr4allephi@gmail.com*

*ORcid:https://orcid.org/my-orcid?orcid=0000-0002-2784-3564*

*Contact Number:  +251 913687219*
